# Meniscal Ossicles as micro-CT Imaging Biomarker in a Rodent Model of Antigen-Induced Arthritis: a Synchrotron-Based X-ray Pilot Study

**DOI:** 10.1038/s41598-017-08025-7

**Published:** 2017-08-08

**Authors:** Sandro Donato, Serena Pacilè, Federico Colombo, Chiara Garrovo, Simeone Dal Monego, Paolo Macor, Giuliana Tromba, Stefania Biffi

**Affiliations:** 1 Elettra - Sincrotrone Trieste S.C.p.A., Basovizza, Trieste Italy; 2 Department of Physics, University of Trieste and INFN, Trieste, Italy; 3 Department of Engineering and Architecture, University of Trieste, Trieste, Italy; 4 Department of Life Sciences, University of Trieste, Trieste, Italy; 5 Institute for Maternal and Child Health, IRCSS Burlo Garofolo, Trieste, Italy; 6 BioInformatic Services CBM S.c.r.l., Basovizza, Trieste Italy

## Abstract

It is increasingly recognized that early detection of bone erosion plays an important role in the overall evaluation of rheumatoid arthritis and in the choice of the correct treatment approach. Since an appropriate use of imaging biomarkers in preclinical settings offers the prospect of smaller and optimized sample size, in the present study we define an anatomical imaging biomarker that could be objectively measured from micro-CT imaging data as an indicator of bone erosion in arthritis process. The well-characterized antigen-induced arthritis (AIA) model in rats was used. The animals were divided into 2 groups: arthritic disease control and arthritic having been administrated with the tumor necrosis factor alpha-blocking agent (Humira). Rats were sacrificed in the acute phase of AIA; peripheral blood and synovial tissue were collected for assessment of arthritis. *Ex vivo* micro-CT tomography of knee joints was performed at the Elettra synchrotron light source (Trieste, Italy). Overall, results from this study suggest that use of high-resolution micro-CT analysis coupled with meniscal ossicles bone parameters quantification provide a powerful combination to enhance data interpretation and assessment of disease-modifying drugs in an animal model of arthritis.

## Introduction

Rheumatoid arthritis (RA) is an autoimmune disease that causes chronic inflammation of synovial tissue infiltrated by T, B and dendritic cells and macrophages producing pro-inflammatory cytokines and autoantibodies that lead to cartilage degradation, bone erosion and damage to the surrounding ligaments^1^. A substantial progress has been made over the past 15 years in RA treatment and the development of biological agents targeting molecules or cells involved in the pathogenesis of RA have dramatically changed its prognosis. Moreover, the staging of the disease remains an unmet clinical need in order to guarantee an early diagnosis; in particular, the progression of the inflammatory process to bone destruction remains a turning point and its early evaluation represents an important parameter in the diagnosis and following disease progression and effectiveness of the therapy^2^.

The characterization of the progression of RA in human is challenging because of limited clinical assessment tools and poor accessibility of disease tissue in the early phase and trough disease progression. In this context, animal models of RA have contributed greatly to the overall knowledge of principal pathogenic factors that causes bone and cartilage destruction, thus leading to important advances in medical products development^3^. Indeed, animal models remain a very important tool for preclinical screening of new therapeutics in pharmaceutical development^4^.

High-resolution and highly sensitive preclinical imaging technologies have evolved considerably and are becoming critical tools to image structural, functional and molecular information in living animals^5^. Computed Tomography (CT), which is extensively used in bone and joint disorders providing excellent spatial resolution and good contrast for bone imaging, is useful to assess structural assessment of mineralized structures^6^. New CT imaging modalities such as high resolution peripheral quantitative computed tomography (HR-pQCT) are promising for giving insight in the microarchitecture and geometry of the bone.

In the present study we applied micro-CT to evaluate morphological changes of the knee joint in a acute model of antigen-induced arthritis (AIA) and meniscal ossicles have been investigated as a potential preclinical imaging biomarker.

## Materials and Methods

### Animals

Wistar rats were obtained from a colony kept in the animal house at the University of Trieste. Male animals weighing 230–260 g were used in this study. The experiments were performed in compliance with the guidelines of the European and Italian laws and were approved by the Italian Ministry of University and Research and by the University Institutional Committee. All treatments were performed with the animals under total anesthesia that was induced with 20 mg/Kg of Zoletil (Virbac) and 5 mg/Kg Xilazina.

### Study design

The arthritis was induced and monitored as previously described^7, 8^. Briefly, the animals (n = 6) were injected with 2 intradermal injections of 150 μg of methylated bovine serum albumin (mBSA) in 200 μl of sterile saline and Freund’s complete adjuvant (both from Sigma). Fourteen days after the second injection, joint inflammation was induced by intra-articular administration of mBSA (100 μg in 100 μl of saline) into the right knee joint. Saline was injected into the left knee joint, which served as a control. Two hours after mBSA challenge, a group of animals (n = 6) were treated intravenously with 300 μg of Humira. The swelling of the right and left knees, which reflect arthritis development, was monitored using a caliper. Rats were euthanized 3–7 days after challenge. Peripheral blood, synovial liquid and synovial tissue were collected for assessment of arthritis, as previously described^7, 8^. To avoid air leakage, alterations and movements during the X-ray examination, samples were then embedded in a 1% agarose gel inside 1.5 ml tubes.

### Synchrotron radiation-based analysis

#### Acquisition

All data sets were acquired at the SYRMEP beamline of the Elettra synchrotron light source (Trieste, Italy), which is especially designed for medical applications and analysis of biological samples^9^. To exploit the spatial coherence of the X-ray source, scans were performed in propagation-based phase contrast modality using the entire white-beam provided by the source, filtered with 1.5 mm of Silicon, and resulting with an average energy of 25 keV. The detecting system consisted in a sCMOS chip based camera, placed 15 cm away from the sample, and coupled with a LSO scintillator (10 µm on 150 µm of YbSO substrate) and a high numerical aperture optic with pixel size set to 2 µm. For each sample we collected 2400 projections over 360°, using the extended field of view mode, with exposure time of 600 ms/projection. The resulting scanned volume was approximately 100 mm^3^. A single distance phase retrieval pre-processing algorithm was applied on the projections (δ/β = 300), prior to the reconstruction procedure based on the standard Filtered Back Projection approach^10^.

#### Post-processing

Post processing phase was done with Avizo® 9.2 imaging software. To evaluate bone focal erosion in the sesamoid bones, these anatomical regions were selected by manually drawn regions of interest. The stack of 2D slices was filtered with a median filter to reduce image noise, and then segmented using auto thresholding based on the Otsu criterion^11^. For the description of bone morphometry, we selected a minimum set of parameters^12^: (i) bone volume fraction (BV/TV, bone volume per total volume), (ii) bone surface density (BS/TV, bone surface per total volume) and (iii) specific bone surface (BS/BV, bone surface per bone volume). BV represents the segmented bone volume (only voxels that belong to ossicles were considered), BS the bone surface computed by triangulation of the object surface, and TV is the total volume obtained from the bone volume filling holes inside the labeled ossicles. Bone parameters are then evaluated using the appropriate Avizo module.

### Statistics

Results from at least three independent experiments are reported as the means ± SD and analyzed for statistical significance by the unpaired Welch Two Sample t-test. A p-value of less than 0,05 was considered significant.

## Results

**Figure 1:**
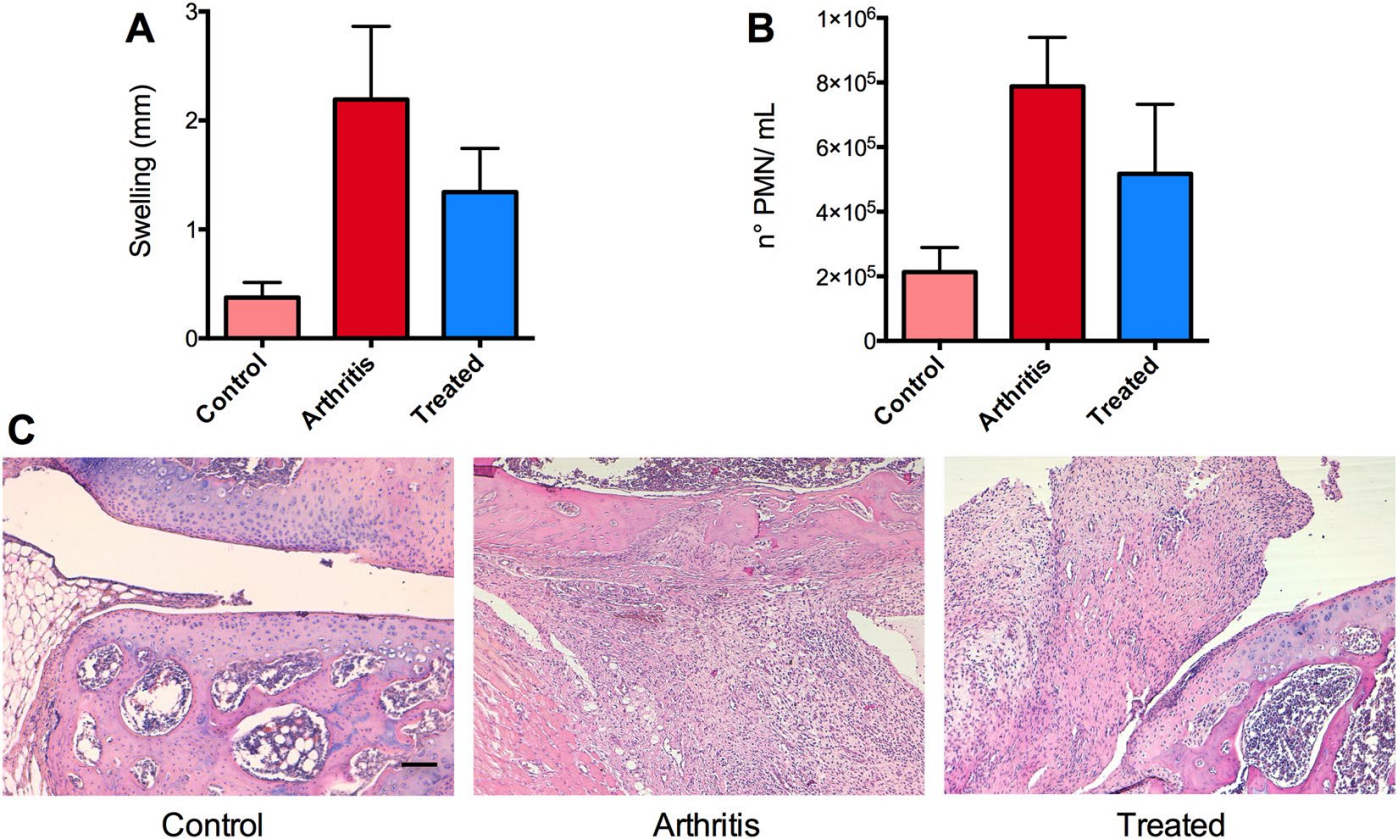
Effect of Humira on the development of antigen-induced arthritis in rats. Two groups of rats were injected intravenously with Humira (300 μg - Treated) or saline (Arthritis) after intra-articular administration of mBSA to induce arthritis. Contralateral joints represent Control group. Animals were euthanized and examined for joint swelling (**A**) and polymorphonuclear neutrophil (PMN) counts in synovial lavage fluids (**B**). Values are the mean +/− SEM of 6 rats per group. Tissue damages (**C**) was also visualized by histological analysis. In the control image we can appreciate a healthy cartilage and synovial tissue, while arthritis samples show iperplastic synovial tissue associated with a huge infiltration of inflammatory cells and a severe cartilage degradation. Tissue of Humira-treated animals show a reduced thickness of the synovial membrane with a decreased infiltration of inflammatory cells and a preserved cartilage. The scale bar is the same for all images: 50 μm.

**Figure 2:**
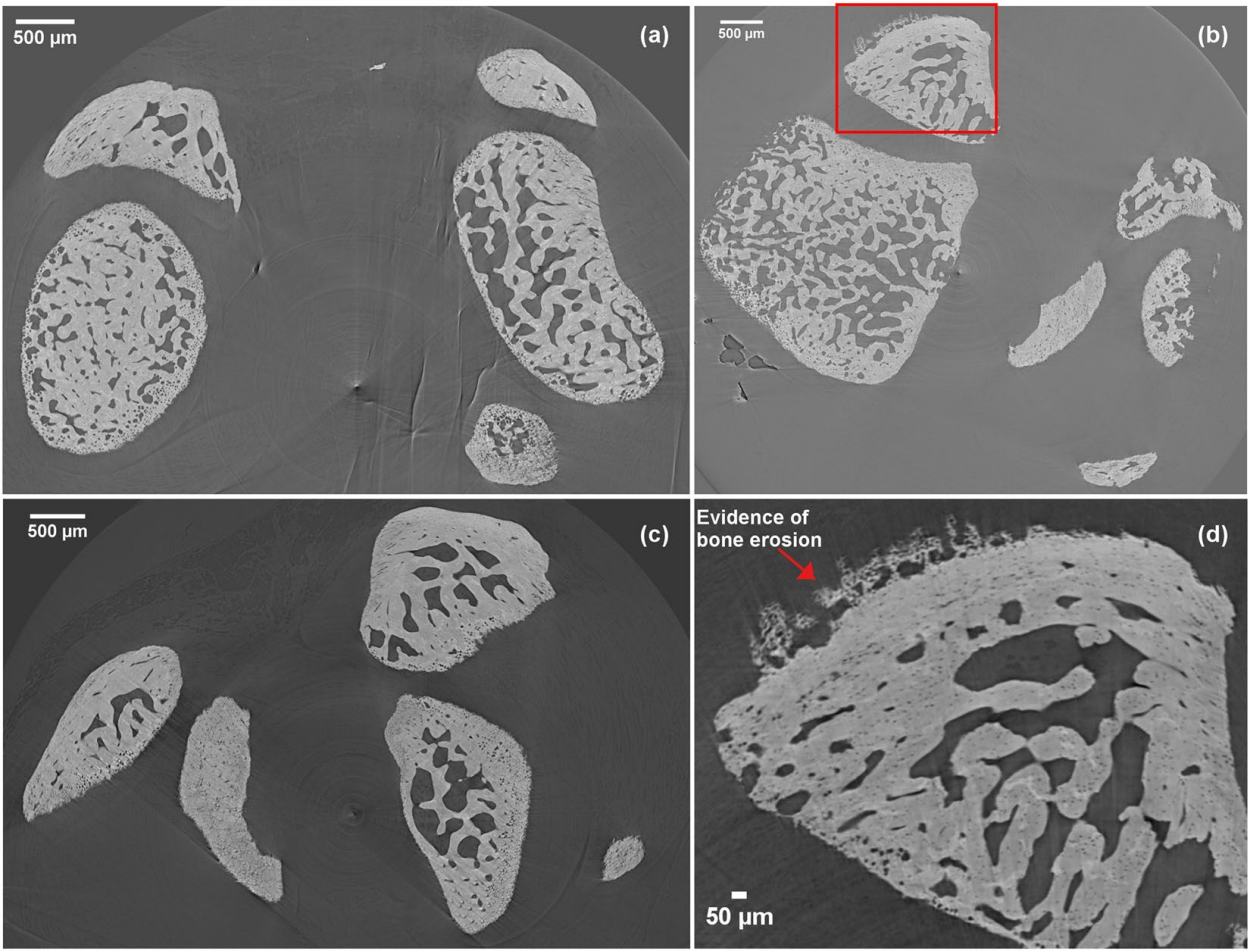
Comparison of representative micro-CT slices of knee joints from Antigen-Induced Arthritis (AIA) and AIA+Humira rats. (**a**) Contralateral joint; (**b**) Injected knee joint; (**c**) Injected knee joint after systemic treatment with Humira; (**d**) Enlarged detail showing bone erosion. Micro-CT slices from the injected joints showed evidence of bone erosion indicated by loss of bone density along the edge of periarticular surface (red arrow). AIA+Humira rats displayed a much smoother joint surface resembling that observed in the contralateral control knee.

The primary aim of this study was to explore the potential of meniscal ossicles as imaging biomarker to monitor bone erosion associated to acute immune-complexes induced inflammation. To this end, we evaluated the bone status of knee joints in an AIA rat model by using an *ex vivo* high-resolution micro-CT imaging approach. Intra-articular methylated bovine serum albumin (mBSA) injection was done in mBSA-sensitized rats and whole-articular imaging was performed acquiring micro-CT images 3–7 days post-injection, which correspond to the pick of the acute phase of inflammation. In this RA model, there is no large inter-animal variability and local intra-articular induction allows the possibility to develop a monoarthritis model, with the contralateral knee joint acting as an internal control. The development of immune-complexes induced inflammation was mainly documented by evaluating the swelling of the articulation and polymorphonuclear leukocytes (PMNs) influx in comparison to control rat (Fig. 1). As shown in Fig. 2, the *ex vivo* high-resolution micro-CT images revealed marginal erosions and loss of bone density along the edges of articular surfaces in mBSA-treated joints as compared to controls, which showed preservation of articular bone. These data were in line with PMNs count and swelling degree as indicators of the inflammatory process into the synovial membrane. Histological analysis shown in Fig. 1 confirmed tissue damage and bone erosion/infiltration in this phase of AIA, described both by swelling/PMN and micro-CT analysis.

In parallel, since recent studies on tumor necrosis factor alpha antagonist (anti-TNF) therapy in RA patients have found that erosive damage may “heal” in some RA patients treated with anti-TNF^13, 14^, we evaluated the activity of Humira in preventing bone erosion, as a consequence of the inflammatory process. Rats (n = 6/group) were either treated with control vehicle (PBS) or with Humira (300 μg i.v.) 2 hours after mBSA intra-articular challenge. We observed the anti-inflammatory effect of antibody and its capacity to prevent bone erosion; treated joints showed smooth and well-defined surfaces similar to structures obtained in samples from healthy knees (Fig. 2). These results reflect the reduction in inflammation previously evidenced measuring joint swelling and PMN influx and confirmed by morphological images (Fig. 1).

**Figure 3:**
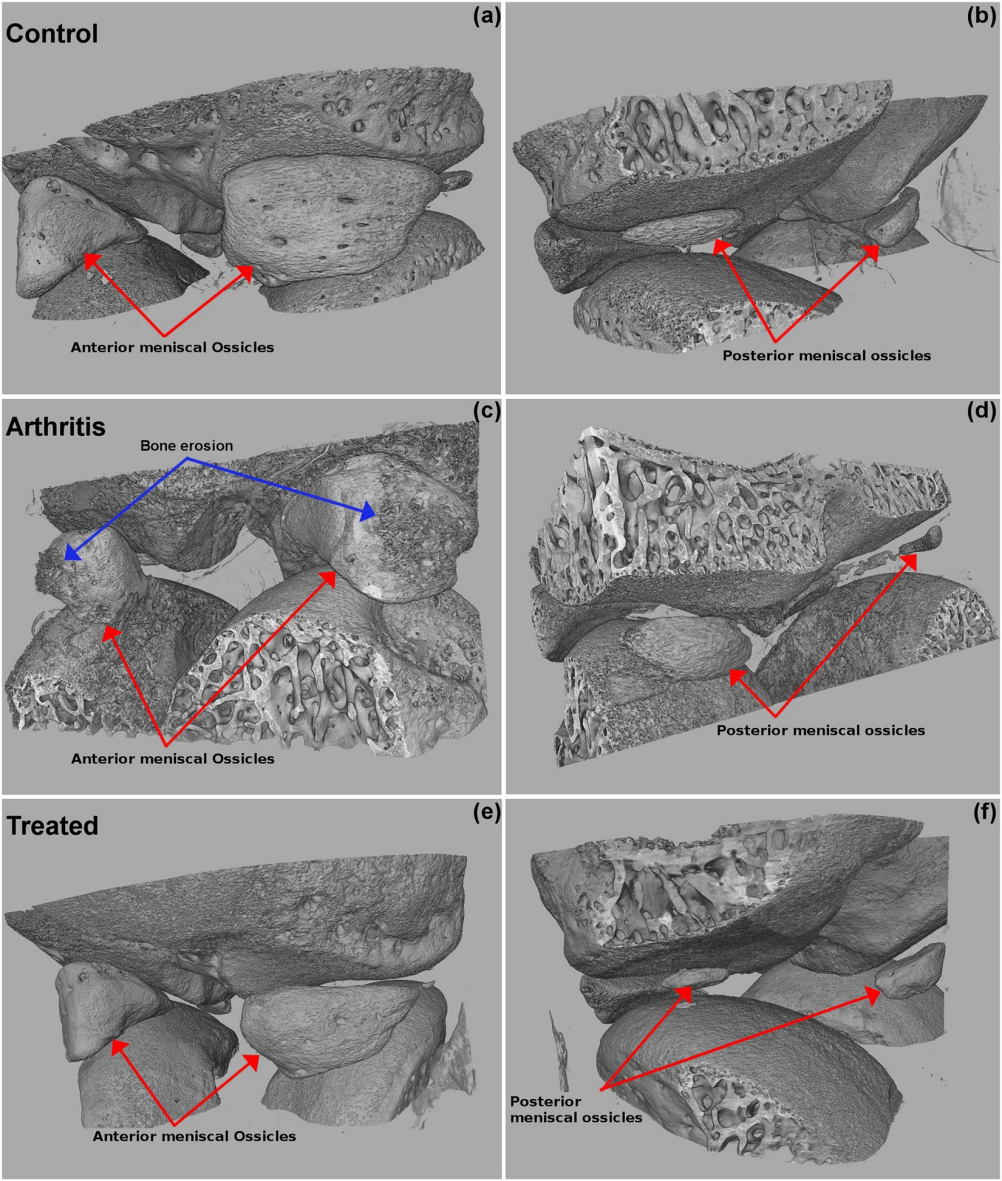
3D structure of meniscal ossicles from image processing of micro-CT data. Representative images of contralateral joint (*control*), injected knee joint (*arthritis*) and injected knee joint after systemic treatment with Humira (*treated*) are shown from anterior view (**a**,**c**,**e**) and posterior view (**b**,**d**,**f**).

**Figure 4:**
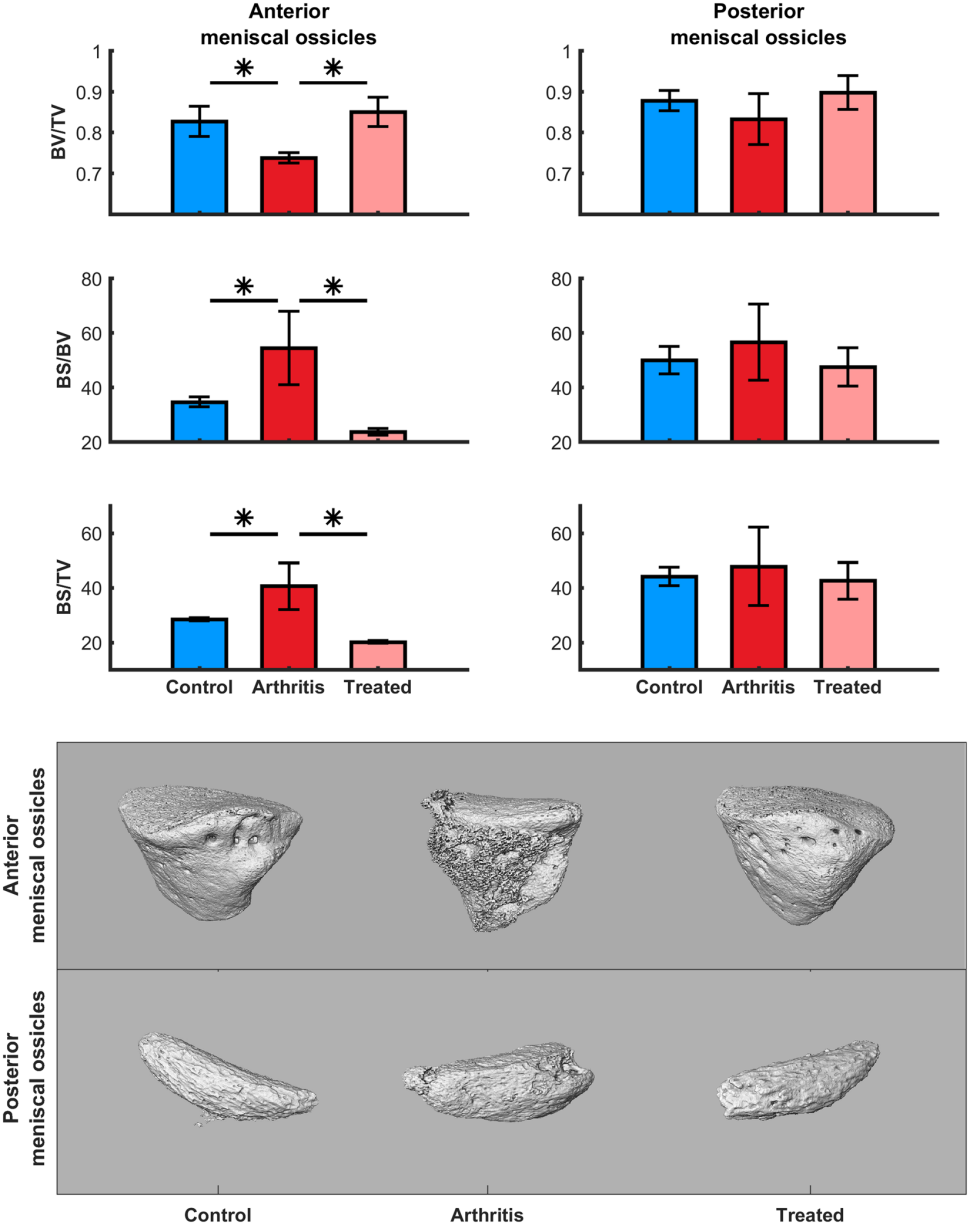
Effect of antigen injection and Humira treatment on the bone parameters of meniscal ossicles. Upper panel shows bone parameters (BV/TV, BS/BV, BS/TV, mm^−1^) analysis at the meniscal ossicles. BV, bone volume; TV, total volume; BS, bone surface. Data were presented as means ± SD, n = 3/group. Lower panel shows representative 3D images of anterior and posterior meniscal ossicles in contralateral joint (*control*), injected knee joint (*arthritis*) and injected knee joint after systemic treatment with Humira (*treated*).

Volumetric evaluation was also performed by means of visual inspection of articular surfaces, which confirmed 2D analysis data. Of note, volume reconstruction of knee joints allowed visualizing meniscal ossicles in 3D within a whole-articulation context at the micrometer resolution range (Fig. 3). Consistently with previous literature describing rat joint anatomy^15^, we observed pyramid-shaped ossicles in the anterior portions, and *lunulae* ossicles in the posterior lateral portions. Since a qualitative analysis of 3D reconstruction showed significant difference between these ossicles, we next quantitatively analysed different bone parameters in order to evaluate the potential ability of meniscal ossicles to serve as a radiological signature for assessing alterations of bone. The bone parameters (BV/TV, bone volume per total volume; BS/BV, bone surface per bone volume; BS/TV, bone surface per total volume) at the posterior meniscal ossicles were not significantly influenced by mBSA challenge and Humira treatments (Fig. 4). Of interest, at the anterior meniscal ossicles, the bone volume fraction (BV/TV) was significantly decreased also in this acute inflammatory phase as compared to controls (n = 3, decrease ~11%, p-value = 0,0032), but recovered after Humira treatment to the same level of the not inflamed joint (Fig. 4). The inflammatory process also caused a statistically significant increase in the parameters quantifying surface density at the anterior meniscal ossicles as compared to controls (n = 3, BS/BV increase ~36%, p-value = 0,0273; n = 3, BS/TV increase ~30%, p-value = 0,0293). In parallel, treatment with Humira was able to abrogate the BS/BV and BS/TV increase (Fig. 4). This effect might be attributed to its ability to prevent the external erosion on the periarticular surfaces and was confirmed by the qualitative analysis of 3D reconstruction (Fig. 3).

## Discussion

Micro-CT in animal models appears to be particularly well suited for high-resolution characterization of the bones and can be used to define quantitative parameters to assess RA in an experimental context, thereby providing more insights on the disease process and on therapeutic protection from bone erosions^16, 17^. However, it is important to note that the results obtained from micro-CT datasets strongly depend on a number of technical issues, including selected volumes of interest (VOI), segmentation, thresholding procedures and scanning resolutions. Among all parameters, definition of a reproducible VOI is a key factor to monitor changes in bone microarchitecture and it may be challenging, due to variability in VOI location, shape and size. In this context, our current findings define an anatomical imaging biomarker that could be objectively marked out as an indicator of arthritis process at bone level. Indeed, in our experimental model we found that several bone parameters were altered in the anterior meniscal ossicles, closely linked to the presence of the disease, thereby providing a means to monitor the bone damage in a defined anatomical region. In particular, by performing a targeted analysis of meniscal ossicles as structures of interest, the necessary presumption of topological equivalence across datasets is satisfied, and no subjective decision must be taken about the VOI setting, thus improving objectivity and reproducibility. Moreover, producing an accurate non-destructive quantitative analysis on the entire target volumes, this method is not operator-dependent and can be easily applied systematically on a high number of samples.

Based on extensive previous experience^7, 8, 18, 19^, the AIA was chosen because represents a model of acute inflammation with early stage of bone damages, the possibility to follow contralateral joint as a not inflamed control and because imaging analysis are possible at known phase of disease. Since it is increasingly recognized that early detection of bone erosion play an important role in the understanding of disease pathology, the ability of micro-CT analysis to identify bone erosion at acute phase of inflammation is of interest. Moreover, our current findings underline that anatomical factors render some areas of juxta-articular bone more susceptible to erosion. Indeed, in our experimental model we found anterior meniscal ossicles to be affected by mBSA challenge, while the posterior meniscal ossicles appeared unaltered by the early phase arthritic process. Due to the key role of synovitis in trigger bone erosions, typically observed at the sites synovium comes into direct contact with bone, the ability of Humira to counteract the inflammation of synovium induced by inflammatory process likely represents a plausible mechanism of the decreased bone erosion in anterior meniscal ossicles of Humira-treated AIA animals. Of note, our results are consistent with the evidence that early intervention with anti-rheumatic therapy effectively reducing the burden of synovitis is the most effective approach for the prevention of bone erosions.

Overall, results from this study suggest that use of high-resolution micro-CT analysis and quantification of meniscal ossicles bone parameters provide powerful combination to enhance data interpretation and assessment of disease modifying medicine in AIA model. Meniscal ossicles as imaging biomarker could provide a rapid and quantitative screening tool for preclinical evaluation of therapeutics: it has the potential to provide results over a shorter time frame, to increased objectivity and to make it easier the setting of blinded readers. Lastly, we need to consider that although a method for capturing radiologic signatures in RA conditions that allow comparison of human pathology with animal models would be of great value in translational research, the differences between human and rodent ossicles would make this comparison challenging. Indeed, human meniscal ossicles, unlike their rodent counterparts, appear to have a prevalence of 0.15%^20^. Moreover, the functional-anatomical basis of the meniscal ossicles recognized in clinical settings remains poorly understood^21^.
